# Nutrient digestibility, hindgut metabolites and antioxidant status of dogs supplemented with pomegranate peel extract

**DOI:** 10.1017/jns.2017.34

**Published:** 2017-07-13

**Authors:** Tony Jose, Ashok K. Pattanaik, Sunil E. Jadhav, Narayan Dutta, Shalini Sharma

**Affiliations:** Niche Area of Excellence in Clinical Nutrition, Division of Animal Nutrition, ICAR Indian Veterinary Research Institute, Izatnagar-243 122, India

**Keywords:** Antioxidants, Dog nutrition, Gut health, Pomegranate peel extract, GSH, reduced glutathione, PPE, pomegranate peel extract

## Abstract

The present study assessed the effect of dietary supplementation of pomegranate peel (methanolic) extract (PPE) on the nutrient digestibility, faecal fermentative metabolites and antioxidant status of dogs. Six medium-sized dogs of mixed breeds were allocated to two groups in a replicated cross-over design and fed a basal diet with and without supplementation of PPE at 50 mg/kg body weight. Each of the experimental periods consisted of 30 d, with a 21 d washout period in between. Results indicated that PPE supplementation led to no significant changes in nutrient digestibility except a trend of improvement (*P* = 0·070) in crude fibre digestibility. PPE supplementation significantly (*P* *<* 0·001) influenced faecal pH, ammonia and lactate production, indicative of positive effects of PPE polyphenols. PPE supplementation further increased faecal SCFA concentration accompanied by a reduction (*P* *<* 0·01) in branched-chain fatty acids. PPE supplementation also improved (*P* < 0·05) indices of erythrocytic antioxidants, namely reduced glutathione, catalase, glutathione peroxidase and glutathione *S*-transferase together with an improvement in lipid peroxidation. Overall, it can be concluded that dietary supplementation with PPE at 50 mg/kg body weight had a positive impact on hindgut fermentation and antioxidant status in dogs, thereby demonstrating its potential as a gut health-promoting nutraceutical.

Phytochemicals are an emerging group of functional compounds which are being increasingly studied for their potential gut health-promoting effects. Microbes in the hindgut convert complex plant polyphenols to simple bioactive compounds, that may make an impact both locally and systemically on gut health^(^^1^^)^. Polyphenol supplementation from various sources^(^^2^^,^^3^^)^ including pomegranate^(^^4^^)^ have been found to improve growth of probiotic organisms in the hindgut, together with a reduction in different pathogens, leading to higher production of fermentative metabolites which are beneficial for health.

Pomegranate (*Punica granatum*) is a fruit of the family Punicaceae, and is rich in polyphenols possessing anti-inflammatory, antioxidant, anti-bacterial and anticancerous properties^(^^5^^)^. Ellagitannins characterised by ellagic acid, gallic acid and punicalagin are the predominant phenolics of the pomegranate^(^^6^^)^. Total phenolic contents of pomegranate peel extract (PPE) is 10-fold higher than that of its pulp extract. Of variously prepared PPE and pomegranate seed extracts, the methanolic extract of pomegranate peel has reportedly shown the highest antioxidant activity^(^^7^^)^. Pomegranate polyphenols have also exhibited positive effects on health-promoting bacteria (*Bifidobacterium breve* and *B. infantis*) alongside inhibitory effects on pathogenic bacteria (clostridia and *Staphyloccocus aureus*)^(^^5^^)^. Supplementation of pomegranate polyphenols has resulted in altered metabolism of gut bacteria, leading to an increased production of SCFA^(^^4^^)^.

Most of the reports on the gut health-modifying ability of pomegranate extracts are based on *in vitro* studies. Indeed, very few *in vivo* evaluation reports are available in the scientific literature. Furthermore, there are no reports on the evaluation of PPE in dogs. The present study investigated the effects of PPE supplementation on nutrient utilisation, erythrocytic antioxidant development and hindgut fermentation in dogs.

## Materials and methods

All experiments were approved by the Institute Animal Ethics Committee as per the ‘Committee for the Purpose of Control and Supervision of Experiments on Animals’ guidelines. The animals were housed in the institute kennel with adequate hygiene measures, and were allowed exercise for 1 h each during morning and evening hours in the adjacent open area.

Pomegranate peels, collected from a local juice shop in a single lot, were sun-dried and subsequently ground to a fine powder using a laboratory mill (SM 100; Retsch GmbH). Crude polyphenolic extract was prepared using methanol as per Althunibat *et al*.^(^^8^^)^. The powder was stored at 4°C in an air-tight container.

### Animals, diets and feeding

A total of six medium-sized female healthy dogs of mixed breeds (aged about 1 year; 18·09 (se 0·27) kg body weight) were randomly divided into two equal groups (control and PPE) and were used in a replicated cross-over design involving two periods. Each period consisted of 30 d with a 21 d washout period in between the two testing periods. The dogs had an ideal body condition score and were declared healthy by a veterinarian based on medical history, physical examination, complete blood count and serum biochemistry. The control group dogs were fed the basal diet with no supplementation. The PPE group dogs were fed the basal diet supplemented with methanolic polyphenolic extract at a dose of 50 mg/kg body weight. The basal diet was prepared by pressure cooking the following ingredients: rice, wheat, Bengal gram, soyabean meal, skimmed milk and vegetable oil, with the nutrient profile matching National Research Council recommendations for adult maintenance^(^^9^^)^ (Supplementary Table S1). The amount of food for individual dogs was calculated to meet the maintenance energy requirement (kcal = 130 × kg body weight^0·75^; kJ = 544 × kg body weight^0·75^), and was offered in two equal portions in the morning (09.30 hours) and evening (19.30 hours) in feeding bowls. Food intake was monitored daily. A 1–4-point scale (1 = ate entire meal without hesitation, 4 = refused to eat) was adopted for subjective assessment of experimental diet palatability^(^^10^^)^ by a single observer.

### Digestibility trial and faecal analyses

A digestibility trial was conducted for 4 d between days 26 and 29 of experimental feeding during each testing period. This involved quantification of daily food intake and faecal excretion to determine the apparent total tract digestibility of nutrients^(^^11^^)^. Representative samples of food offered and residues left, if any, were collected from each meal, pooled animal-wise and used for DM determination and subsequent proximate analyses^(^^12^^)^. All the faeces excreted by individual dogs were collected immediately after voiding and preserved under refrigeration until sampling time at the end of the 24 h period. The faeces were homogenised, and aliquots were collected for determination of pH measurement and fermentative end-products. The pH of the faecal samples was measured using a digital pH meter (Eutech Instruments). Subsequently, the faecal samples were processed for estimation of ammonia, lactate, SCFA and branched-chain fatty acids^(^^13^^)^.

### Blood collection and analyses

Blood samples were collected from the cephalic vein of dogs in two separate vials (with heparin and acid dextrose citrate; ACD) for estimating erythrocyte antioxidants and blood metabolites before and after each feeding period and processed as detailed elsewhere^(^^14^^)^. Blood samples collected in ACD were centrifuged at 2000 ***g*** for 20 min after being refrigerated for 2 h to harvest the erythrocytes. The buffy coat layer was removed and erythrocytes were washed with cold PBS solution. The packed erythrocytes obtained after three washings were used for estimation of reduced glutathione (GSH). Erythrocytic suspension was prepared by mixing equal volumes of erythrocytes and a normal saline solution. This suspension was used for Hb estimation. Exactly 0·1 ml of erythrocytic suspension was mixed with 0·9 ml of stabilising solution (containing EDTA and β-mercaptoethanol) to form haemolysate. The samples were kept at −70°C and used for an antioxidant assay involving GSH, lipid peroxidation, catalase, superoxide dismutase, glutathione peroxidase and glutathione *S*-transferase as described elsewhere^(^^15^^)^.

The heparinised blood samples were used for separation of plasma by centrifugation (3000 ***g*** for 15 min) and stored at –20°C until analysed for glucose, total protein and albumin, TAG, total cholesterol, LDL-cholesterol and HDL-cholesterol using diagnostic kits (Span Diagnostics).

### Statistical analyses

Statistical analysis was carried out with IBM SPSS Statistics 20.0 (SPSS Inc.). Blood metabolite and antioxidant data were analysed adopting a pre-test–post-test method. Changes in the post-test means were compared by ANCOVA adjusted for pre-test means. Results are expressed as means and standard errors. Significance was declared at *P* ≤ 0·05.

## Results and discussion

### Intake and digestibility of nutrients

The mean daily DM intake was 294·5 (se 6·8) and 295·3 (se 5·3) g for the control and PPE groups, respectively, exhibiting no difference between them. The palatability score remained similar between the two groups, indicating an absence of any adverse influence of food acceptability. As such, the mean daily intakes of crude protein, ether extract, crude fibre and N-free extract were similar between the two dietary groups. Further, the digestibility of DM (79·1 (se 1·4) *v*. 79·5 (se 1·8) %), crude protein (80·8 (se 1·1) *v*. 82·0 (se 1·5) %) and ether extract (84·4 (se 1·4) *v*. 84·8 (se 1·8) %) remained uninfluenced by dietary PPE supplementation. However, digestibility of fibre (32·5 (se 1·5) *v*. 39·6 (se 3·2) %) tended to be higher (*P* = 0·070) with PPE supplementation. The range of DM digestibility values (about 80 %) observed in the present study are comparable with earlier reports in dogs fed home-prepared diets^(^^11^^,^^16^^)^. The same is true for the digestibility values observed for other nutrients including crude protein, ether extract and N-free extract. The observed trend for improved fibre digestibility could have been due to the potential impact of PPE polyphenols on improving hindgut fermentation.

### Faecal fermentation metabolites

**Fig. 1.**
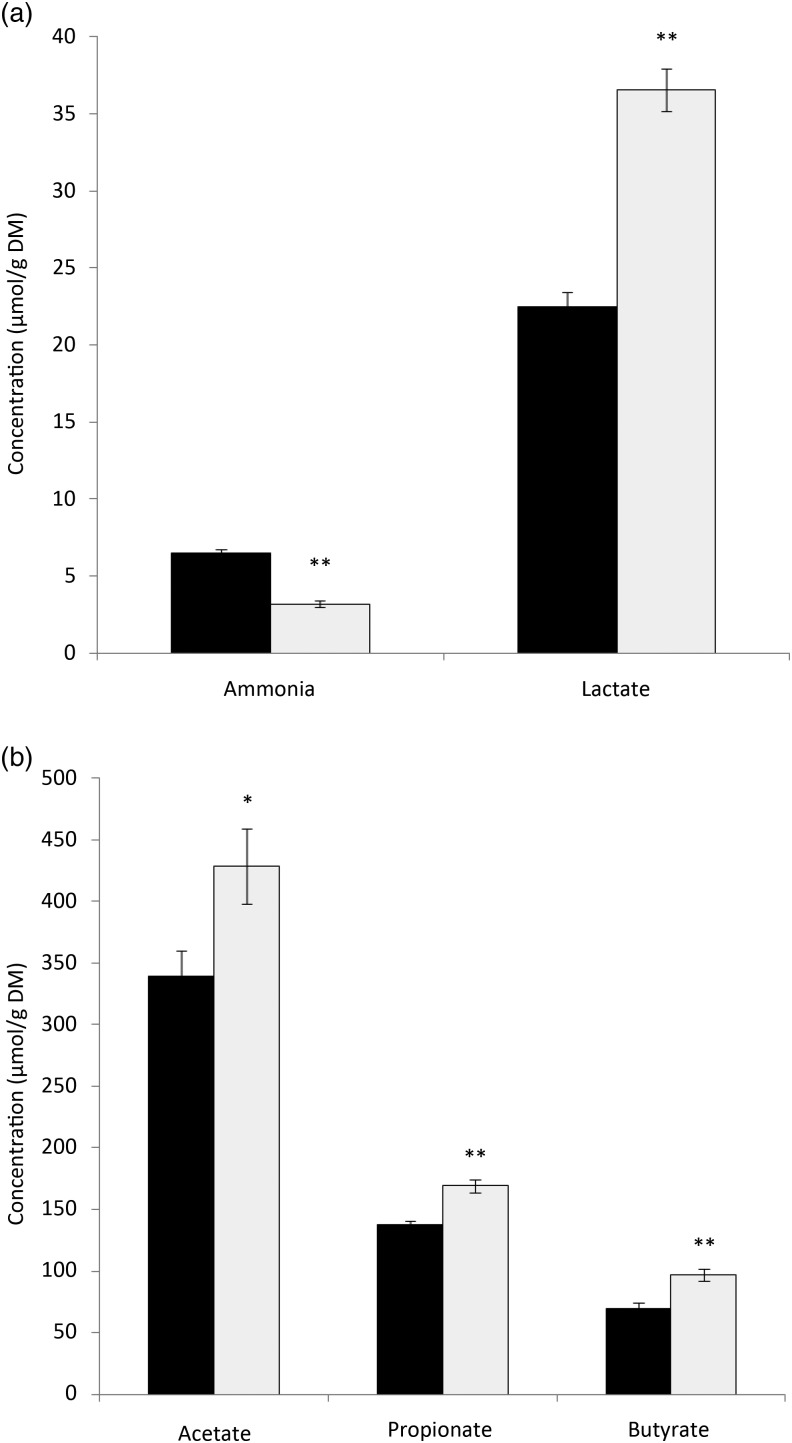
Effect of dietary supplementation of pomegranate peel extract (PPE; 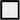) on the concentrations of (a) ammonia and lactate and (b) SCFA in the faeces of dogs. Values are means (*n* 6), with standard errors represented by vertical bars. Mean value was significantly different from that for the control (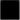) period: * *P* < 0·05, ** *P* < 0·01.

The data on faecal analysis are presented in Fig. 1. The faecal ammonia concentration was significantly lower in the PPE group when compared with the control. Conversely, faecal lactate levels were increased significantly in the PPE group as compared with the control group. There was a significant increase in the concentration of faecal acetate, propionate, and butyrate in PPE-supplemented dogs. Among the faecal branched-chain fatty acids, isovalerate did not differ between the groups; however, there was a significant reduction in valerate concentration in the PPE group (21·88 (se 1·45) µmol/g DM) as compared with the control group (29·23 (se 1·49) µmol/g DM). Isobutyrate was non-detectable in the faeces of PPE-treated dogs. Butyrate is regarded as the most important SCFA in the large intestine because it stimulates the proliferation of epithelial cells, mucus release and water and mineral absorption^(^^17^^)^. Some of the beneficial impacts of polyphenols on gut health include enhanced production of SCFA, especially butyrate, which enhances colorectal epithelial health, besides preventing the development of colorectal cancer^(^^18^^)^. Therefore, the present results imply that PPE is effective in modulating microbial fermentation and increasing the production of desirable fermentation end-products. The faecal alterations due to PPE supplementation resulted in a significant reduction in faecal pH (5·93 (se 0·04) *v*. 6·63 (se 0·07)) as compared with controls. A decreased faecal pH due to the greater production of SCFA and lactic acid during fermentation is an indicator of optimal hindgut health, as low pH prevents the growth of harmful microbes and improves the absorption of nutrients.

### Erythrocytic antioxidants and blood metabolites

The erythrocytic antioxidant status of the dogs (Table 1) indicated that PPE supplementation decreased lipid peroxidation of erythrocytes. Except for superoxide dismutase, all the other measures of the antioxidant indices, including antioxidant enzymes such as catalase, glutathione peroxidase and glutathione *S*-transferase and the small molecule antioxidant, GSH, showed increases due to dietary PPE supplementation. Earlier results of a rat study using pomegranate fruit rind extract reported a similar increase in superoxide dismutase, catalase, GSH and glutathione peroxidase levels, indicating improved antioxidant status^(^^19^^)^. The antioxidant potency of pomegranate extracts has previously been correlated with their phenolic contents. In fact, the methanolic extract of pomegranate peel has been reported to show the highest antioxidant activity *in vitro* among the extracts prepared from the seed and peel using ethyl acetate, methanol and water^(^^7^^)^. In another study comparing the antioxidant potential of juice, peel, and seed oil of pomegranate, the highest values were recorded for the peels^(^^20^^)^. Among the blood metabolites, plasma levels of glucose, TAG and LDL-cholesterol showed a reduction accompanying an increased HDL-cholesterol in PPE-fed dogs in conformity with the reported hypoglycaemic^(^^21^^)^ and hypolipidaemic^(^^22^^)^ role of polyphenols. The plasma total proteins, albumin and globulin levels, however, remained unchanged.

**Table 1.** Erythrocytic antioxidant measurements in two study periods before (day 0; pre-test) and after pomegranate peel extract (PPE) supplementation (day 30; post-test) (Mean values with their standard errors)

	Day 0				Day 30				
	CON		PPE		CON		PPE		
Parameters	Mean	se	Mean	se	Mean	se	Mean	se	P*
Lipid peroxidation (nmol MDA/mg Hb)	3·41	0·12	3·37	0·13	4·13^b^	0·21	3·84^a^	0·04	0·006
Reduced glutathione (nmol/mg Hb)	3·97	0·18	4·27	0·26	4·09^a^	0·14	4·54^b^	0·07	0·039
Catalase (U/mg Hb)	15·10	0·74	16·02	1·06	13·64^a^	0·76	17·40^b^	0·73	0·006
Superoxide dismutase (U/mg Hb)	65·83	6·60	67·84	3·26	65·18	3·19	67·09	1·86	0·475
Glutathione peroxidase (U/mg Hb)	14·71	1·30	13·42	0·70	16·80^a^	1·19	20·11^b^	1·47	0·003
Glutathione *S*-transferase (U/mg Hb)	5·63	0·26	5·71	0·50	4·13^a^	0·52	6·09^b^	0·89	0·043

CON, control; MDA, malondialdehyde.

* Significance between CON and PPE periods following 30 d of PPE supplementation using ANCOVA adjusted for pre-test means (*n* 6).

Overall, results obtained in this preliminary study have demonstrated the potential role of PPE as a gut health-promoting nutraceutical. However, the low sample size of the study might have affected the outcome. Further studies using a larger sample size are warranted to conclusively validate the present findings.

### Conclusions

Dietary supplementation of PPE at 50 mg/kg body weight had a positive impact on gut health and erythrocytic antioxidant indices in medium-sized breed dogs without any apparent influence on nutrient digestibility, thereby supporting its potential as a gut health-promoting nutraceutical.

## Supplementary material

For supplementary material accompanying this paper visit http://dx.doi.org/10.1017/jns.2017.34. click here to view supplementary material
