# Supplementary material for: Nutrient digestibility, hindgut metabolites and antioxidant status of dogs supplemented with pomegranate peel extract
Source: J Nutr Sci. 2017 Jul 13;6:e36. doi: 10.1017/jns.2017.34 (PMC5672305; doi:10.1017/jns.2017.34)
Supplement: Supplementary file 1 [file S2048679017000349sup001.doc]

**Supplementary Table S1. Ingredient and proximate composition of the basal diet*† fed to the dogs**

| Ingredients | Level |
| --- | --- |
| Ingredient composition (%, fresh basis) | |
| Rice | 19.0 |
| Wheat | 22.2 |
| Soyabean meal | 34.5 |
| Bengal gram | 11.0 |
| Skimmed milk powder | 5.0 |
| Vegetable oil | 6.0 |
| Dicalcium phosphate | 1.3 |
| Calcium carbonate | 1.0 |
| *Chemical composition* | |
| Dry matter (DM; %) | 21.10 |
| (% DM basis) | |
| Organic matter | 95.71 |
| Ether extract | 6.32 |
| Crude protein | 22.57 |
| Crude fibre | 4.99 |
| Nitrogen-free extract | 61.84 |
| Crude ash | 4.29 |
| Metabolisable energy (kJ/g) ‡ | 14.61 |

*Pressure cooked at 15 psi for 10 min.

†Additionally supplemented with a trace mineral supplement (provided per kg of diet: Mn: 14.2 mg; Fe: 110 mg; Cu: 9 mg; Co: 1.8 mg; Zn: 150 mg; I: 1.6 mg; Se: 0.3 mg) and a vitamin supplement (provided per kg of diet: Vitamin A: 11000 IU; Vitamin D: 910 IU; Vitamin E: 57.5 IU; Vitamin K: 0.65mg; thiamin: 7.56 mg; riboflavin: 11.89 mg; pantothenic acid: 18.50 mg; niacin: 93.16 mg; pyridoxine: 6.60mg; biotin: 12.42 mg; folic acid: 1,142.10 mg; Vitamin B_12_: 164.87 mg).

‡Calculated as per AAFCO (Association of American Feed Control Officials): ME = 35·564 kJ ME/g fat + 14·644 kJ ME/g CP + 14·644 kJ ME/g nitrogen-free extract.
